# An era of biological treatment in systemic lupus erythematosus

**DOI:** 10.1007/s10067-017-3933-x

**Published:** 2017-12-12

**Authors:** Jing He, Zhanguo Li

**Affiliations:** Department of Rheumatology & Immunology, Peking University People’s Hospital, 11 Xizhimen South Street, Beijing, 100044 China

The management of active SLE is challenging due to the heterogeneous nature of the disease and lack of specific treatment. Current therapy of active SLE primarily relies on corticosteroids and immunosuppressants [1]. Complete remission is the ultimate goal in SLE treatment, but it is not common in daily practice [2]. The routine therapy of corticosteroid and immunosuppressant is only effective in a portion of patients and associated with substantial adverse effects including infections, osteoporosis, and cardiovascular disorders [3–5]. Therefore, there is an unmet need for new therapies with better efficacy and less adverse effects.

A better understanding of the mechanisms underlying the pathogenesis of SLE has led to rapid development of targeted biological treatments that modulate various aspects of the immune response. Currently, some novel drugs have appeared in SLE patients. There are also promising phase II, III trials targeting B cell, T cell, cytokine, and other molecules.

In the most encouraging B cell targeting, belimumab and rituximab have been used in clinic. The fully humanized monoclonal antibody against soluble trimeric B cell activating factor (BAFF), belimumab, has been approved for the treatment of SLE in Europe and the USA. However, clinical trial showed only 14% higher response rate of belimumab (SRI = 58%) in SLE patients compared to placebo (44%) at week 52 [6]. The CD20 targeting rituximab is considered as an attractive therapeutic target, and shown by a number of publications [7–9]. In a prospective study, rituximab showed promising efficacy in corticosteroid sparing [10]. However, both EXPLORER study of rituximab in nonrenal SLE and LUNAR study in lupus nephritis failed to achieve their primary endpoints [11, 12]. It is no doubt to us that rituximab is effective in SLE, but patient stratification based on clinical features is needed in treatment or in trials. In addition, epratuzumab, a humanized anti-CD22 antibody, showed a decreased CD19^+^ B cell count and favorable clinical response in the EMBLEM phase II trials and well tolerated [13]. But, in the EMBODY phase III clinical trials, treatment with epratuzumab did not show improvements in response rates compared to placebo group (39.8 vs 34.1%) [14]. It appears that further study is required to examine the effects of anti-CD22 antibody in SLE. CD40L could be another novel effective biological treatment for SLE. If CD40L blocker binds to CD40 on the surface of B cells and leads to IgG class switching, then the production of high affinity autoantibodies may be inhibited. Recently, a randomized, double-blind, multicenter phase I trial of dapirolizumab pegol (a polyethylene glycol conjugated anti-CD40L Fab’ fragment) showed 46% patients achieved BICLA (vs 14% placebo), and no serious adverse event occurred [15]. However, due to the small number of patients, further evaluation of this new biologic is required to address its efficacy and safety.

Recently, in a proof of concept trial, we have shown that low-dose IL-2 was efficient and tolerated in active SLE [16]. An SRI response was seen in 34/38 patients (89.5%) at week 12. Resolution of clinical activity was observed in multiple domains, including rash, alopecia, arthritis, fever, leukopenia, and thrombocytopenia. No severe adverse events were observed. There were significant reductions of proteinuria and autoantibodies titers accompanied by increased levels of C3 and C4. It is suggested by several studies that low-dose IL-2 can rebalance aberrant function of the immune system, through promoting Treg-mediated effect and inhibiting Tfh- and Th17-related pathogenic responses [16–20]. Meanwhile, CD8 T cell and NK cell response is enhanced upon this treatment. It is likely that low-dose IL-2 improves the immune response against infection in SLE.

In addition, it has been well documented that high serum IFNα and IFN gene expression signature were seen in SLE patients with active disease [21, 22]. Sifalimumab (anti-IFNα mAb), rontalizumab (humanized IgG1 anti-IFNα antibody), and anifrolumab (anti-interferon receptor 1 (IFNAR1)) showed promising results in phase II and III trials [23–27]. In a phase II study of sifalimumab, the SRI-4 response index was statistically superior in the treatment groups than placebo: 56.5–58.3% in sifalimumab groups vs. 45.4% in the placebo group. However, the data were not impressive as expected with just a slightly increased response. More infection occurred in patients using the study drug [24]. Its safety and efficacy is under study by an ongoing phase III trial. Furthermore, IL12/23 pathway is active in SLE. IL12/23 inhibitor (ustekinumab) was effective in a phase II study, showing that 60% of patients in ustekinumab group had an SRI-4 response vs 31% in placebo at week 24 [28].

Recent years, much attention has been on abatacept, a fusion protein comprised of CTLA-4 (cytotoxic T lymphocyte antigen) combined with the Fc portion of human IgG1 (CTLA-4-Ig). It has been used to inhibit T lymphocyte and retard progression of lupus nephritis in murine models of disease [29–31]. A number of CTLA-4 studies have been focused on SLE patients [32–34], but no significant improvements were observed clinically [31, 32]. However, abatacept did show evidence of biologic activity (reduces anti-dsDNA antibodies, increases C3 concentrations) and was well tolerated in patients with active lupus nephritis. Unfortunately, it did not achieve primary end points [32–34].

Finally, studies have shown that mesenchymal stem cells (MSCs) and T cell vaccine (TCV) are effective and safe in SLE. These are strategies potential in SLE therapy in the future [35–37].

Taking together, anti-Blys, anti-CD20, low-dose IL-2, MSCs, and TCV have been used clinically. Anti-CD22, Interferon-α, CTLA-4-Ig, and some other biologics are in ongoing clinical trials. It is clear that a new era is on the horizon for more targeted therapies for SLE, which will be largely dependent on collaboration of researchers and rheumatologists.
